# Salivary Osteopontin as a Potential Biomarker for Oral Mucositis

**DOI:** 10.3390/metabo11040208

**Published:** 2021-03-30

**Authors:** Enikő Gebri, Attila Kiss, Ferenc Tóth, Tibor Hortobágyi

**Affiliations:** 1 Department of Dentoalveolar Surgery and Dental Outpatient Care, Faculty of Dentistry, University of Debrecen, Nagyerdei krt. 98, H-4032 Debrecen, Hungary; gebri.eniko@dental.unideb.hu; 2 Department of Hematopoietic Transplantation Centre, Faculty of Medicine, University of Debrecen, Nagyerdei krt. 98, H-4032 Debrecen, Hungary; akiss@med.unideb.hu; 3 Department of Biomaterials and Prosthetic Dentistry, Faculty of Dentistry, University of Debrecen, Nagyerdei krt. 98, H-4032 Debrecen, Hungary; ferenc.toth@dental.unideb.hu; 4 Institute of Pathology, Faculty of Medicine, University of Szeged, Állomás utca 1, H-6725 Szeged, Hungary; 5 MTA-DE Cerebrovascular and Neurodegenerative Research Group, Department of Neurology, Faculty of Medicine, University of Debrecen, Nagyerdei krt. 98, H-4032 Debrecen, Hungary; 6 Institute of Psychiatry Psychology and Neuroscience, King’s College London, De Crespigny Park, London SE5 8AF, UK

**Keywords:** autologous peripheral stem-cell transplantation (APSCT), oral immunity, oral mucositis, osteopontin, salivary biomarkers

## Abstract

Osteopontin (OPN), a multifunctional phosphoglycoprotein also presents in saliva, plays a crucial role in tumour progression, inflammation and mucosal protection. Mucosal barrier injury due to high-dose conditioning regimen administered during autologous and allogeneic peripheral stem cell transplantation (APSCT) has neither efficient therapy nor established biomarkers. Our aim was to assess the biomarker role of OPN during APSCT, with primary focus on oral mucositis (OM). Serum and salivary OPN levels were determined by ELISA in 10 patients during APSCT at four stages of transplantation (day −3/−7, 0, +7, +14), and in 23 respective healthy controls. Results: There was a negative correlation between both salivary and serum OPN levels and grade of OM severity during APSCT (*r* = −0.791, *p* = 0.019; *r* = −0.973, *p* = 0.001). Salivary OPN increased at days +7 (*p* = 0.011) and +14 (*p* = 0.034) compared to controls. Among patients, it was higher at day +14 compared to the time of admission (day −3/−7) (*p* = 0.039) and transplantation (day 0) (*p* = 0.011). Serum OPN remained elevated at all four stages of transplantation compared to controls (*p* = 0.013, *p* = 0.02, *p* = 0.011, *p* = 0.028). During APSCT elevated salivary OPN is a potential non-invasive biomarker of oral mucositis whereas the importance of high serum OPN warrants further studies.

## 1. Introduction 

Saliva is one of the most important pillars of oral immunity and a special mirror of the body’s defence mechanisms [1,2,3,4]. As a rich reservoir of proteins, especially glycoproteins, electrolytes, lipids and other molecules with several biological functions, saliva plays a pivotal role in the maintenance of oral homeostasis and mucosal integrity [5]. Changes in its constituents offers an active field for researchers to discover novel and useful biomarkers (‘salivaomics’) [6]. Saliva and its samplings have several advantages compared to other body fluids such as being non-invasive, painless, cost-efficient for diagnosis with possibility of self-collection which is particularly advantageous in a pandemic period [7]. Osteopontin (OPN) has been identified in saliva as a protein involved in mucosal barrier integrity and antimicrobial defence with relevance to oral cavity pathologies including neoplasia [8].

OPN is a multifunctional, chemokine-like, sialic-acid rich phosphoglycoprotein, classified as a member of the Small Integrin-Binding Ligand *N*-linked Glycoprotein (SIBLING) family [9]. It plays a pivotal role in tumour development, progression, inflammation and mucosal protection impacts on cell survival, proliferation and invasion. OPN is expressed by many cell types such as immune, neural, epithelial and endothelial cells, fibroblasts and secreted in body fluids including blood, cerebrospinal fluid and saliva. OPN gene expression is modulated by several factors such as cytokines (e.g., IL-1ß, IL-6), hormones (e.g., oestrogen, progesterone (P4)) and growth factors [10]. Overexpression of OPN in several cancers such as breast cancer, malignant haematological diseases or oral squamous cell carcinoma (OSCC) [11,12] predicts poor overall survival, suggesting its role as a prognostic biomarker [10]. OPN is an effective regulator of the hematopoietic stem cell homeostasis and neutrophil migration [13]. It plays a crucial role in several non-neoplastic processes, including graft-versus-host disease (GVHD) after allogeneic hematopoietic stem cell transplantation [14]. Its role in mucosal defence, especially against viral pathogens [15] and in tissue destruction with subsequent repair process, is also essential [16].

Hematopoietic stem cell transplantation (HSCT) is a routinely used, increasingly successful and widely applied therapeutic tool in the treatment of malignant hematologic disorders [17]. On the other hand, mucosal barrier injury (MBI) is an inevitable and occasionally life-threatening complication of the administered conditioning regimen during HSCT, which may affect the entire digestive tract. It is a disease of multifactorial etiopathogenic origin with several patient and treatment related risk factors such as: (i) the nature of the conditioning regimen, (ii) Gram negative translocation of the resident microflora, (iii) changes in the quality and quantity of salivary constituents (iv) duration of neutropenia and the time of engraftment formation; (v) very young or old age; (vi) ethnicity; (vii) poor oral hygiene; (viii) low body mass; (ix) genetic predispositions, causing and modulating the injury of the protective mucosal barrier [18,19]. Mucositis is a result of a complex pathobiological process, which affects 60% to 100% of transplanted patients, decreases quality of life, prolongs hospital stay and may predispose to several serious complications, including GVHD [18]. It has neither efficient therapy nor established biomarkers [20]. Several cytostatic treatments used prior to and high-dose intensive conditioning therapies administered during HSCT enhance the risk of secondary malignancies, especially oral cancers [21].

**Figure 1:**
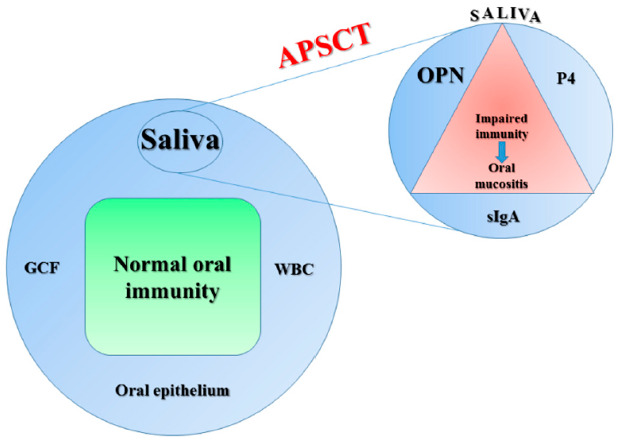
Pillars of oral immunity (on the left) and salivary biomarkers for oral mucositis (on the right) besides impaired immunity during autologous peripheral stem cell transplantation (APSCT) based on our previous findings. Saliva, oral epithelium, gingival crevicular fluid (GCF), and white blood cells (WBC) are key components of oral immunity, with a prominent role of saliva [1]. Changes in salivary constituents (e.g., glycoproteins-immunoglobulin A (IgA), hormones-progesterone (P4)) could be good predictors and biomarkers of several oral and systemic diseases including oral mucositis, as we reported earlier in more details [2,3,4].

Our aim was to examine the changes in oral immunity together with the role of OPN in mucosal protection during autologous peripheral stem cell transplantation (APSCT) and identify novel biomarkers for oral mucositis and subsequent oral pathologies as a result of high-dose intensive cytostatic treatment (Figure 1).

## 2. Results 

### 2.1. Serum OPN Levels

**Figure 2:**
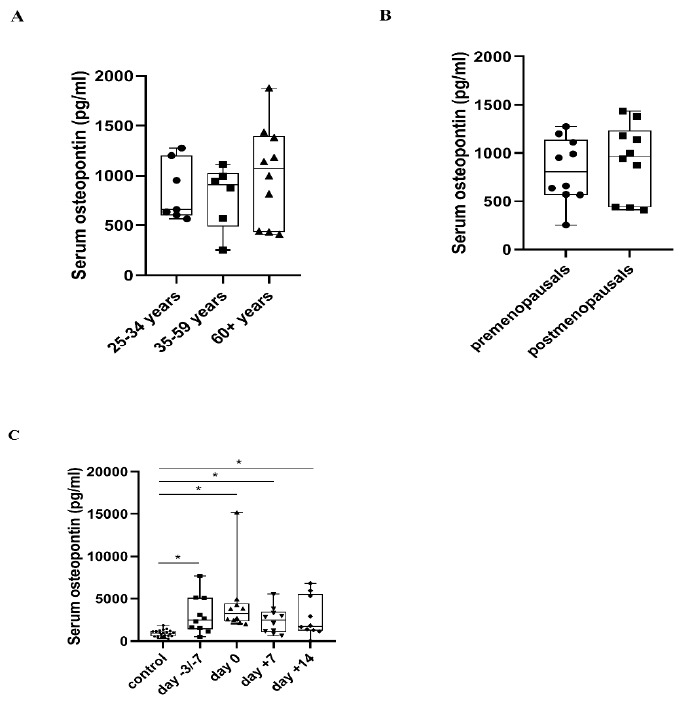
Serum osteopontin (OPN) concentration in healthy controls in relation to age (**A**) and hormonal status of women (**B**), and in patients at four stages of autologous peripheral stem cell transplantation (APSCT) (**C**). The start of conditioning therapy (day −3 in patients with multiple myeloma receiving melphalan and day −7 in patients with lymphoma receiving BEAM conditioning) was defined as the first day of samplings. Values are expressed as sample means. The small black configurations (dots, triangles, squares, etc.) are individual data points, and the box plots denotes the data mean. Error bars represent the standard deviations which describe average difference between the data points and their mean. (* *p* ˂ 0.05).

There was no significant difference in serum OPN levels regarding either age or pre- and postmenopausal hormonal status in the control group. Considerable overexpression could be observed during APSCT at all four stages of transplantation (day −3/−7, day 0, day +7, day +14) compared to the control group (*p* = 0.013, *p* = 0.02, *p* = 0.011, *p* = 0.028) (Figure 2).

### 2.2. Salivary OPN Levels

**Figure 3:**
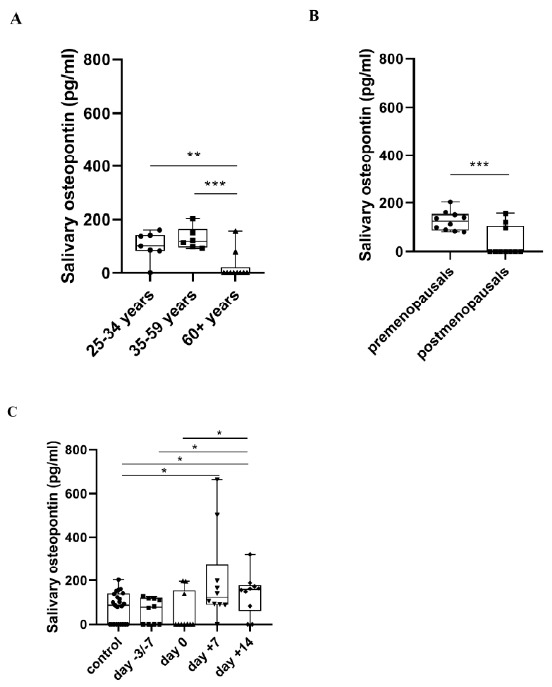
Salivary osteopontin (OPN) concentration in healthy controls in relation to age (**A**) and hormonal status of women (**B**), and in patients at four stages of autologous peripheral stem cell transplantation (APSCT) (**C**). The start of conditioning therapy (day −3 in patients with multiple myeloma receiving melphalan and day −7 in patients with lymphoma receiving BEAM conditioning) was defined as the first day of samplings. Values are expressed as sample means. The small black configurations (dots, triangles, squares, etc.) are individual data points, and the box plots denotes the data mean. Error bars represent the standard deviations which describe average difference between the data points and their mean. (* *p* ˂ 0.05, ** *p* ˂ 0.01, *** *p* ˂ 0.001).

Salivary OPN level was significantly lower in the elderly control group compared both to the middle-aged and the young adults group (*p* = 0.001, *p* = 0.01), while there was no difference between the middle-aged, and the young adults group (*p* = 0.305). Premenopausals showed significantly higher salivary OPN level than postmenopausal controls (*p* = 0.001). There was no significant difference in salivary total protein concentration neither in relation to age nor to hormonal status (Supplementary Figure S1A,B). OPN/total protein concentration ratio (i.e., normalized OPN concentrations) in the elderly control group was lower compared both to the middle-aged and the young adults groups (*p* = 0.003, *p* = 0.012). There was no significant difference in the middle-aged group compared to the young adults group (*p* = 0.945) and it was lower in the postmenopausals compared to the premenopausals (*p* < 0.001), in concert with the changes of absolute (non-normalized) OPN levels (Supplementary Figure S2A,B). These indicate that decrease in OPN is not due to a decrease in protein content in general. During APSCT there was a significant increase at day +7 and day +14 in salivary OPN levels compared to the control group (*p* = 0.011, *p* = 0.034) and at day +14 compared to the day of admission (day −3/−7) and transplantation (day 0) (*p* = 0.039, *p* = 0.011) (Figure 3).

### 2.3. UWS Flow Rate 

**Figure 4:**
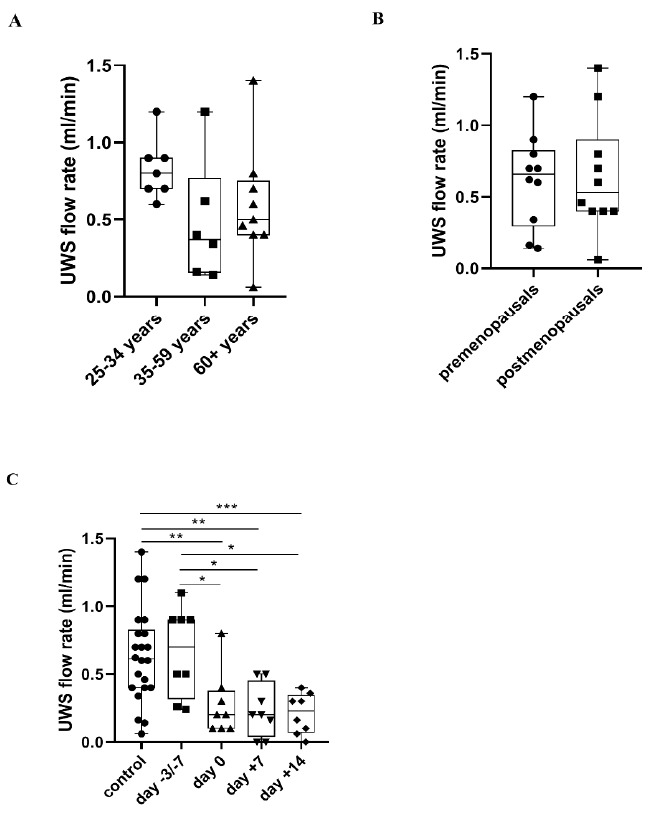
Changes in unstimulated whole saliva (UWS) flow rate in healthy controls in relation to age (**A**) and hormonal status of women (**B**), and during autologous peripheral stem cell transplantation (APSCT) (**C**) compared to controls. The start of conditioning therapy (day −3 in patients with multiple myeloma receiving melphalan and day −7 in patients with lymphoma receiving BEAM conditioning) was defined as the first day of samplings. Values are expressed as sample means. The small black configurations (dots, triangles, squares, etc.) are individual data points, and the box plots denotes the data mean. Error bars represent the standard deviations which describe average difference between the data points and their mean. (* *p* ˂ 0.05, ** *p* ˂ 0.01, *** *p* ˂ 0.001).

There was no significant difference in UWS flow rates neither between the three age groups nor between the pre-and postmenopausals. No significant difference was observed in UWS flow rate at the day of admission (day −3/−7) compared to the control group whereas the amount of UWS decreased significantly at day 0, day +7 and day +14 compared both to the control group and the day of admission (*p* = 0.008, *p* = 0.004, *p* = 0.001, *p* = 0.012, *p* = 0.012, *p* = 0.012) (Figure 4).

Tables S3–S5 in the Supplementary online materials summarize the serum, salivary OPN levels and UWS values (mean, standard deviation and number of cases) in relation to age, sex and hormonal status both in healthy controls and patients during APSCT.

### 2.4. Serum CRP Levels

**Figure 5:**
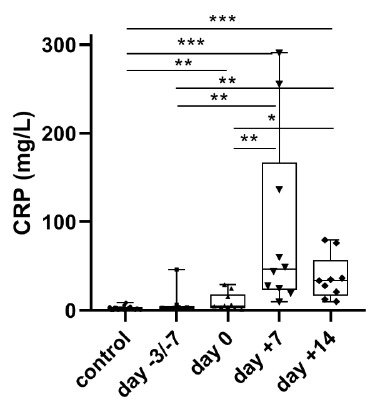
Changes in serum CRP levels at four stages of autologous peripheral stem cell transplantation (APSCT). The start of conditioning therapy (day −3 in patients with multiple myeloma receiving melphalan and day −7 in patients with lymphoma receiving BEAM conditioning) was defined as the first day of samplings. Values are expressed as sample means. The small black configurations (dots, triangles, squares, etc.) are individual data points, and the box plots denotes the data mean. Error bars represent the standard deviations which describe average difference between the data points and their mean. (* *p* ˂ 0.05, ** *p* ˂ 0.01, *** *p* ˂ 0.001).

Serum CRP levels increased significantly at day +7 and day +14 compared to the controls, the admission day and the day of transplantation (*p* = 0.0002, *p* = 0.0001, *p* = 0.005, *p* = 0.008, *p* = 0.005, *p* = 0.011). There was also a significant difference in serum CRP levels between the controls and the day of transplantation (*p* = 0.0026) (Figure 5).

### 2.5. Correlation Analyses

There was a significant negative correlation between both salivary and serum OPN levels and grade of OM during APSCT (*r* = −0.791, *p* = 0.019; *r* = −0.973, *p* = 0.001). Salivary P4 level [3] showed a significant positive correlation with salivary OPN level in the postmenopausal control group (*r* = 0.944, *p* = 0.001). Neither pre-transplantational serum LDH level, nor pre-treatment time showed a significant correlation with serum OPN levels at the day of admission (day −3/−7). Nor did we find any significant correlation between OPN levels and the returned amount of stem cells, stem cell viability, viable cell count and amount of mononuclear cells (MNC) at the day of transplantation (day 0). CRP level showed a significant positive correlation with serum OPN level only at day +14 of the four stages of transplantation (*r* = 0.700, *p* = 0.036).

## 3. Discussion 

Injury of the protective mucosal barrier is one the most severe and often life-threatening complications of hematopoietic stem cell transplantation, contributing to mortality. It may predispose to further serious complications, such as disseminated infections, veno-occlusive disease or GVHD [18]. The combination of short-term, intensive cytostatic treatments occasionally complemented with total body irradiation (TBI) during HSCT and long-term chemotherapies as immunosuppressive treatments prior to transplantation enhances the incidence of secondary malignancies [21]. Consequently, regular post-transplant dental care and identification of novel biomarkers indicating oral cancers and rejection are sorely needed. OPN, with its broad range of biological activities, and saliva, as a rapidly developing field of biomarker research, may be sufficient tools in this contest [22,23,24].

### 3.1. Serum OPN Levels in Healthy Controls and APSCT Patients

OPN is expressed in several tissues and body fluids, and plays a pivotal role in both physiological and pathological processes [14]. We have scarce relevant data of serum OPN levels in healthy controls. In spite of this, our age-specific results are consistent with those of Cristaudo et al., who did not find any significant correlation in serum OPN levels between ages within the control group in his study, either [25] (Figure 2A). As mentioned earlier, OPN gene expression is modulated by several factors, including hormones [10] via their receptors like oestrogen receptor-alpha (ER-α) [26]. Several studies have described that postmenopausal women have higher serum OPN levels than premenopausals [27,28]. Nevertheless, other studies have observed that in postmenopausal women serum OPN levels were inversely associated with bone mineral density (BMD) [28]. As OPN upregulates osteoclast motility and bone resorption, it has a pivotal role in osteoporosis [28,29]. Mohamed et al. also confirmed that postmenopausal women with osteoporosis with or without any complication have significantly higher serum OPN levels than healthy postmenopausals [30]. This may suggest that elevated OPN concentrations in postmenopausal women primarily arise from osteoporosis. In our study there was no difference in serum OPN levels between pre-and postmenopausals, a finding accounted for by the fact that, in addition to physiological age-dependent bone resorption, our postmenopausal controls, had no or well-controlled osteoporosis by antiresorptive agents (Figure 2B). 

OPN plays a key role in hemopoietic stem cell (HSC) regulation within the endosteal HSC niche and hemopoiesis in the bone marrow microenvironment [13,31]. OPN assures stem cell homing by attracting HSC [13]. It regulates trans-marrow migration of transplanted HSC and as a negative regulator of HSC proliferation, it ensures the maintenance of HSC quiescence and the size of the HSC pool [13]. Overexpression of OPN during aberrant hemopoiesis is one of the most common features of several hematologic malignancies, presumably as a consequence of the suppression of normal residual HSC proliferation [13,31,32,33]. Recent studies have described that serum OPN levels were increased in patients with MM and this correlated with disease progression and bone destruction [34,35]. Flamant et al. observed that OPN expression had increased rapidly during progression of chronic myeloid leukaemia (CML), while remarkably declined in remission [36]. There is an association of higher OPN expression with a more aggressive variant of lymphoma [37]. Several other studies have confirmed the prognostic significance of OPN both in acute myeloid and lymphoid leukaemia [38,39,40,41]. In our study, serum OPN level showed marked overexpression at all four stages of transplantation compared to the control group. This indicates that OPN is a reliable biomarker for the presence of haematological malignancies also during APSCT (Figure 2C).

### 3.2. Salivary OPN Levels in Healthy Controls and APSCT Patients

OPN is also produced by salivary glands (SG) and its expression differs between humans and rodents [42], demonstrating species-dependent variations [43]. OPN expression is localized in the luminal membrane of acinar cells with a difference in OPN isoforms between the major SGs [42] in mice, whereas in humans it is primarily expressed by the ductal epithelium and the mucinous acinar cells [44]. There is a significant decrease in salivary flow rate in old age with a decrease in the number of acinar cells, accompanied by an increase in the number of adipocytes and fibroblasts [45]. These changes suggest that the lower salivary OPN level in the oldest age group in our study (Figure 3A) is a consequence of these physiological processes. In addition, our results correspond to other studies which described a significant decline in salivary OPN levels in elderly individuals [46]. Previous reports have confirmed that saliva composition shows hormone-related changes. Oestrogen-beta (ER-ß) expression both in acinar and ductal cells in the minor and major salivary glands emphasizes the pivotal role of sex steroid hormones in regulation of saliva secretion [47]. In our study salivary OPN level was significantly lower in postmenopausal controls than in premenopausals. Furthermore, a significant positive correlation was observed between salivary OPN and P4 levels in postmenopausal control women (Figure 3B). Also progesterone modulates OPN gene expression [48], influences cytokine expression and up-regulates OPN production [49]. OPN increases in the mid- to late secretory phase of the cycle, parallel with P4. These findings suggest that the significant decrease of salivary OPN in postmenopausal controls found in our study is primarily not an age-related, but principally a hormone-dependent change. Association of OPN expression with hormonal changes in saliva warrants further research, as it could serve as a suitable tool for screening endocrine abnormalities.

The role of OPN is also essential in both innate and adaptive immune responses, tissue destruction and subsequent repair process [16]. It is expressed by several immune cells (e.g., neutrophils, macrophages/microglia, T-lymphocytes, dendritic and natural killer cells) and controls immune cell functions such as monocyte adhesion, migration, differentiation and phagocytosis [10]. Elevated levels of OPN in body fluids is a good indicator of progression in numerous inflammatory diseases of the cardiac and nervous systems and intestines [50,51]. During inflammation OPN is secreted by T-lymphocytes, macrophages, fibroblasts and myofibroblasts, contributing to granulation tissue formation and connective tissue remodelling around the ulcerated epithelium [52,53]. OPN secreted by epithelial cells is required in the maintenance of epithelial barrier integrity and promotes the transition from innate to adaptive immune response with initiation of repair [16]. In our study there was a significant negative correlation between both salivary and serum OPN levels and OM grade during APSCT, highlighting the pivotal role of OPN in mucosal protection. At the same time, significant overexpression could be observed in salivary OPN level at day +7 of transplantation compared to controls, suggesting the biomarker role of salivary OPN in oral mucositis during APSCT. A slightly declining tendency of salivary OPN was observed at day +14 compared to day +7. Parallel with this, a significantly elevated level of salivary OPN was found at day +14 compared to both controls and the day of admission and transplantation. This reflects the effect of APSCT on local oral immunity and stresses the importance of patient follow-up. Zeiser et al. reported a significant correlation of OPN with the diagnosis of chronic GVHD and severity of the disease [22,54,55,56]. On the other hand, Blijlevens et al. found that development of oral mucositis predisposes to GVHD [18]. Based on these finding we assume that salivary OPN could serve as a good predictor of oral GVHD and secondary malignancies of the oral cavity following HSCT (Figure 3C).

### 3.3. Changes of UWS Flow Rate during APSCT

Degenerative histological changes in the salivary glands induced by chemotherapies are well-known contributing to decreases salivary flow rate during APSCT [57]. Ductal dilatation, cyst formation, acinar degeneration and infiltration of inflammatory cells in the minor, while interstitial fibrosis, vacuolization and nuclear degeneration of both acinar and ductal cells in the major salivary glands were demonstrated [58]. These results are in line with our previous report [2] (Figure 4).

### 3.4. Correlations 

Several studies have revealed the negative prognostic significance of OPN with tumour progression and poor survival [59,60].

Serum LDH level increases in many pathological conditions [59]. As a good indicator of the remaining tumour mass it has an important prognostic role [3,61]. However, we did not find a significant difference, which could be due to the smaller sample size in our study.

Serum CRP level was significantly elevated at day +7, when OM was most severe, and at day +14 of transplantation (Figure 5) and it decreased parallel with the resolution of the inflammation in particular OM. A positive significant correlation was found between serum CRP and serum OPN level at day +14, consistent with the key role of OPN in the resolution phase of acute inflammation [16].

We didn’t find any significant correlation between OPN levels and the returned amount of stem cells, stem cell viability, viable cell count and amount of mononuclear cells (MNC) at the day of transplantation (day 0), in spite of the fact that elevation of serum OPN level was the highest at this time point. Tumour lysis as a result of the conditioning regimen could explain it.

Limitation of the study is the relatively small number of patients in the disease group, although it proved to be enough and sufficient to reach strong statistical significance.

## 4. Materials and Methods

### 4.1. Study Population, Ethics and Patient Characteristics

Our prospective study was carried out at the Hematopoietic Transplantation Centre collaborating with the Dental Outpatient Care, University of Debrecen, Hungary including 10 patients who had required and undergone APSCT and 23 respective healthy controls. All subjects gave their informed consent for inclusion before they participated in the study. The study was conducted in accordance with the Declaration of Helsinki, and the protocol was approved by the Regional Institutional Research Ethics Committee, Clinical Centre, University of Debrecen (Ethical license: DE RKEB/IKEB 4948-2018). Inclusion criterion was the presence of malignant haematological diseases requiring APSCT in the patient population, while participants with severe chronic disease (diabetes, autoimmune diseases, acute or chronic inflammatory diseases, etc.) and previous malignancy were excluded from the study in both groups. Average age was 52.20 ± 13.78 years in the patient group, and 52.00 ± 20.00 years in controls, respectively. Three age groups were created based on the literature [62]: young adults (25–34 years), middle-aged (35–59 years) and elderly (60 ≤ years). Male:female ratio was 3:7 in the transplanted group and 3:20 in the controls. All participants (both patients and healthy controls) answered a questionnaire containing data about age, sex, peritransplantation time interval, smoking habits, alcohol consumption, dental rounds and hormonal status. Based on their hormonal status reflected by their answers in our questionnaire (Table S1) women were divided into two groups, pre- and postmenopausal. Reasons for APSCT were Hodgkin lymphoma (HL) in 1 patient, Non-Hodgkin lymphoma (NHL) in 6, and multiple myeloma (MM) in 3 cases. Pre-treatment time (the period between diagnosis and transplantation), was 15 ± 4.9 months in patients with lymphoma and 13.33 ± 11.37 months in the MM group with no significant difference (*p* = 0.9563). Pre-transplantational stage of the disease was complete morphologic remission (CMR) in 7 cases and partial remission (PR) in 2 patients. Conditioning regimens, according to the European Society for Blood and Marrow Transplantation recommendation [63,64], administered prior to transplantation in Hodgkin and Non-Hodgkin lymphoma were as follows: BEAM (BCNU, etoposide, cytosine arabinoside, melphalan) protocol and Adcetris-BEAM in 1-1 case, Rituximab-BEAM in 5 cases, while melphalan in three scenarios in MM (1. high-dose melphalan (≥200 mg/m^2^) 2. melphalan in dose 140 mg/m^2^ and 3. melphalan combined with Rituximab and Bendamustin). OM was classified according to the World Health Organization (WHO) guidelines (Grade 0–4) each day before samplings (0 none; 1 soreness ± erythema; 2 erythema, ulcers, patient can swallow solid food; 3 ulcers with extensive erythema, patient cannot swallow solid food; 4 mucositis to the extent that alimentation is not possible) [65]. All patients received combined antimicrobial prophylaxis (1. levofloxacin 500 mg 2. fluconazole 100 mg and 3. acyclovir 400 mg iv.) with G-CSF during the post-transplantation period of cytopenia. Patients in both groups were free of dental foci (dental calculus, radices, etc.) during APSCT and at the time of samplings. Table 1 and Supplementary Table S2 summarize further details of patients’ demographics.

### 4.2. Serum and Unstimulated Whole Saliva (UWS) Sample Collection

Blood and Unstimulated Whole Saliva (UWS) samplings were performed at the same time point (between 7 a.m. and 8 a.m., taking into consideration the diurnal rhythm of saliva constituents) on specified days of the peritransplantation period as follows: hospital admission and start of the conditional therapy (day −3/−7), transplantation day, before administering the harvested CD34+ stem cells in a stem cell infusion (day 0), the day of usually the deepest point of cytopenia with most severe oral mucositis (day +7), the day of neutrophil and thrombocyte engraftment if insertion and proliferation of stem cells were successful (day +14), based on the protocols described in details previously [2,3,4]. Briefly, blood samples were collected into BD Vacutainer Blood Collection Tubes using a clot activator (BD, Franklin Lakes, NJ, USA), centrifuged at 1200× *g* for 30 min and the serum fractions were stored at −70 °C until further processing within one hour of collection.

UWS saliva was collected for 5 min in pre-disinfected lockable Falcon tubes (Sigma-Aldrich, St. Louis, MO, USA) 1 h after eating, drinking and tooth brushing, following oral cavity rinse with physiological saline solution (B. Braun Mesulgen AG, Germany) for 30 s in participants at rest. Halt Protease Inhibitor Cocktail (Sigma-Aldrich, St. Louis, MO, USA) was added to the UWS samples and after aliquoting; samples were stored at −70 °C until processing.

### 4.3. Detection of Serum and Salivary OPN Levels

Serum and saliva samples stored at −70 °C were thawed at room temperature and centrifuged at 4 °C for 30 min at 1200 rpm and at 4 °C for 10 min at 3000 rpm. Two- and fourfold dilutions of serum and saliva samples, respectively, were used. OPN levels were measured by Human Osteopontin ELISA Kit RAB0436-KT (Sigma-Aldrich, St. Louis, MO, USA) according to the manufacturer’s instructions. Osteopontin concentrations were normalized to total protein concentration determined by BCA protein assay kit (Thermo Scientific, Waltham, MA, USA).

### 4.4. Detection of Serum C-Reactive Protein (CRP) and Lactate Dehydrogenase (LDH) Levels

CRP and LDH levels were determined using electrochemiluminescence immunoassay (ECLIA) (Roche, Basel, Switzerland) according to the manufacturer’s instructions.

### 4.5. Detection of Salivary Progesterone (P4) Levels 

Salivary P4 levels were determined using electrochemiluminescence immunoassay (ECLIA) (Roche, Basel, Switzerland) according to the manufacturer’s instructions and published in our earlier study [3].

### 4.6. Statistical Analysis

Statistical analysis was performed using IBM SPSS22 software (IBM, Armonk, NY, USA).

Kolmogorov-Smirnov test was used to investigate the distribution of data. One-way analysis of variance (ANOVA) and Kruskal-Wallis test was used to investigate the relation of the serum and salivary OPN levels and UWS flow rate at four stages of transplantation compared to the control group and between the three age groups. In case of normal distribution, we compared two groups using independent sample t-test or paired t-test in the continuous variables, whereas in non-normal distribution we applied Mann-Whitney and Wilcoxon tests. Pearson correlation analysis was performed to correlate serum and/or salivary OPN concentrations with oral mucositis grades, CRP, LDH values and the returned amount of stem cells, stem cell viability, viable cell count, amount of mononuclear cells (MNC), while Spearman correlation for P4 levels. *p* < 0.05 was considered significant.

## 5. Conclusions

Our results indicate that salivary OPN is a rather unexplored, promising non-invasive biomarker for several oral pathologies including OM, while serum OPN could serve as a biomarker for the presence of haematological malignancies also during APSCT. Thorough analysis of different OPN isoforms in the context of post-translational modifications such as glycosylation may also provide to clues to the etiopathogenesis of OM, mucosal defence mechanisms against aggressive pathogens and could be a potential target for treatment.

## Figures and Tables

**Table 1:** Demographic and clinical data of participants.

		Patients		Controls	
		(*n* = 10)		(*n* = 23)	
**Average age (years)**		52.20 ± 13.78		52.20	
**Male/female ratio**		3:7		3:20	
**Pretreatment time (months)**		14.5 ± 6.74			
Total patient group		15 ± 4.9			
Lymphoma group (HL/NHL)		13.33 ± 11.37			
MM group					
**Diagnosis**					
Hodgkin lymphoma		1			
Non-Hodgkin lymphoma		6			
Multiple myeloma		3			
**Pretransplantational stage of the disease**					
Complete remission		7			
Partial remission		3			
**Conditioning regimen**					
BEAM		1			
R-BEAM		5			
Adcetris-BEAM		1			
Melphalan (140 mg/m^2^)		1			
Melphalan (≥200 mg/m^2^)		1			
R-Bendamustin-Melphalan		1			
**Pretransplantational LDH level (physiological range: 135–220 U/L)**		277.33 ± 66.97			
**Amount of stem cells (10^6^/body mass kg)**		4.56 ± 1.87			
**Stem cell viability (%)**		87.17 ± 11.73			
**Viable cell count (10^6^/body mass kg)**		3.95 ± 1.57			
**MNC (10^8^/body mass kg)**		5.59 ± 6.11			
**Highest grade of OM (WHO)**					
0		0			
1		4			
2		3			
3		2			
4		1			
**Last status**					
Alive		8			
Dead		2			
**Hormonal status in female (n)**					
Premenopausal		1		10	
Postmenopausal		6		10	
**Salivary progesterone (P4) levels (µg/L)**					
Premenopausal’s	Postmenopausals’			0.34 ± 0.10	0.21 ± 0.03
	day −3/−7	0.24	0.21 ± 0.02		
	day 0	0.20	0.20		
	day +7	1.86	0.56 ± 0.73		
	day +14	0.87	0.84 ± 1.20		

BEAM (BCNU, etoposide, cytosine arabinoside, melphalan); HL (Hodgkin lymphoma); LDH (lactate dehydrogenase); MM (multiple myeloma); MNC (mononuclear cell); NHL (non-Hodgkin lymphoma); OM (oral mucositis); R-BEAM (Rituximab-BCNU, etoposide, cytosine arabinoside, melphalan); R-Bendamustin-Melphalan (Rituximab); WHO (World Health Organization).

## Data Availability

The data presented in this study are available in this article or as supplementary material.

## Supplementary Materials

The following are available online at https://www.mdpi.com/article/10.3390/metabo11040208/s1, Table S1. Data sheet and questionnaire, Table S2. Patient characteristics. Length of hospital stay and total duration of oral mucositis, Table S3. Serum osteopontin concentration in healthy controls in relation to age, sex and hormonal status of women and in patients at four stages of autologous peripheral stem cell transplantation, Table S4. Salivary osteopontin concentration in healthy controls in relation to age, sex and hormonal status of women, and in patients at four stages of autologous peripheral stem cell transplantation, Table S5. Unstimulated whole saliva flow rate in healthy controls in relation to age, sex and hormonal status of women, and in patients at four stages of autologous peripheral stem cell transplantation, Figure S1. Salivary total protein concentration in healthy controls in relation to age (A) and hormonal status of women (B), Figure S2. Salivary normalized osteopontin concentrations in healthy controls in relation to age (A) and hormonal status of women (B).

## Abbreviations

ANOVA	one-way analysis of variance
APSCT	autologous peripheral stem-cell transplantation
BEAM	BCNU, etoposide, cytosine arabinoside, melphalan
BMD	bone mineral density
BMT	bone marrow transplantation
CML	chronic myeloid leukemia
CMR	complete morphologic remission
CRP	c-reactive protein
EDTA	ethylenediaminetetraacetic acid
EBMT	European Society for Blood and Marrow Transplantation
ELISA	enzyme linked immunosorbent assays
ER-α	estrogen receptor-α
ER-ß	estrogen receptor-ß
GCF	gingival crevicular fluid
GVHD	graft-versus-host disease
HSC	hematopoietic stem cell
HSCT	hematopoietic stem cell transplantation
HL	Hodgkin lymphoma
IgA	immunoglobulin A
sIgA	secretory immunoglobulin A
LDH	lactate dehydrogenase
MBI	mucosal barrier injury
MNC	mononuclear cell
MM	multiple myeloma
NHL	non-Hodgkin lymphoma
OM	oral mucositis
OPN	osteopontin
OSCC	oral squamous cell carcinoma
P4	progesterone
PR	partial remission
SG	salivary gland
SIBLING	Small Integrin-Binding Ligand N-linked Glycoprotein
TBI	total body irradiation
UWS	unstimulated whole saliva
WBC	white blood cells
WHO	World Health Organization
