# Supplementary material for: Salivary Osteopontin as a Potential Biomarker for Oral Mucositis
Source: Metabolites. 2021 Mar 30;11(4):208. doi: 10.3390/metabo11040208 (PMC8066152; doi:10.3390/metabo11040208)
Supplement: Supplementary file 1 [file metabolites-11-00208-s001.pdf]

SUPPLEMENTARY MATERIAL ONLINE

Salivary Osteopontin as a Potential Biomarker for Oral Mucositis

Enikő Gebri, Attila Kiss, Ferenc Tóth, Tibor Hortobágyi

Table S1. Data sheet and questionnaire

<table>
<tr><td colspan="3">Name:</td></tr>
<tr><td colspan="3">National Insurance Number:</td></tr>
<tr><td colspan="3">Date of birth:</td></tr>
<tr><td colspan="3">Day of admission:
Day of transplantation:
Day of discharge:
Length of hospital stay (days):</td></tr>
<tr><td colspan="3">1.) How often do you see a dentist?
1. once every six months or more frequently
2. yearly
3. less often
4. only if I have a problem with my teeth</td></tr>
<tr><td colspan="3">2.) Do you smoke?
1. no
2. occasionally
3. regularly; a little
4. a lot; more regularly
5. earlier yes, but not now</td></tr>
<tr><td colspan="3">3.) Do you drink alcohol?
1. no
2. occasionally
3. regularly; a little
4. a lot; more regularly
5. earlier yes, but not now</td></tr>
<tr><td>4.) Did you have any oral mucosal lesion during the previous treatments (e.g. ulcers)?

If so, what kind?</td><td>☐
Yes</td><td>☐
No</td></tr>
<tr><td colspan="3">5.) Oral hygiene status by physical evaluation is:
1. neglected
2. adequate
3. excellent</td></tr>
<tr><td colspan="3">6.) Sialometry:
day -3/-7:
day 0:
day +7:
day +14:</td></tr>
</table>

**7.) pH values**<br>day -3/-7:<br>day 0:<br>day +7:<br>day +14:		
**Hormonal status in women**		
Do you have periods?	☐ Yes	☐ No
If so, do you take oral contraceptives (OAC)?	☐ Yes	☐ No
If not, when was your last period?		
Time elapsed since last period to the day of admission of APSCT:		
Have you ever been pregnant?	☐ Yes	☐ No
If so, how many pregnancies have you had?		
Did you have any gingival problem during pregnancy?	☐ Yes	☐ No
If so, what kind?		
If you are postmenopausal, did you have any gingival or oral complain (ulceration or anything else) during the perimenopausal phase?	☐ Yes	☐ No
If so, what?<br>1. xerostomy<br>2. burning mouth<br>3. pain in the oral cavity<br>4. gingival bleeding<br>5. tooth luxation<br>6. facial pain		
Do you have any gynecological disease?	☐ Yes	☐ No
If so, what is it?		
What kind of hormone therapy do you receive now and how long have you been taking it?		

APSCT (autologous peripheral stem cell transplantation); OAC (oral contraceptives)

Table S2. Patient characteristics. Length of hospital stay and total duration of oral mucositis (OM)

	Length of hospital stay (days)	Total duration of OM (days)	Grade 0 (days)	Grade 1 (days)	Grade 2 (days)	Grade 3 (days)	Grade 4 (days)
**Patient 1**	37	34	1	27	3	4	0
**Patient 2**	22	18	4	8	10	0	0
**Patient 3**	18	15	3	12	3	0	0
**Patient 4**	29	28	1	13	4	6	5
**Patient 5**	34	12	16	12	0	0	0
**Patient 6**	22	21	0	10	4	7	0
**Patient 7**	16	10	6	10	0	0	0
**Patient 8**	21	10	11	10	0	0	0
**Patient 9**	21	-	-	-	-	-	-
**Patient 10**	21	-	-	-	-	-	-
**Mean**	24.1	18.5	5.25	12.75	3	2.125	5
**SD**	6.90	8.75	5.60	5.97	3.34	3.04	0
**N**	10	8	8	8	8	8	8

N (number of cases); OM (oral mucositis); SD (standard deviation)

Table S3. Serum osteopontin (OPN) concentration in healthy controls in relation to age, sex and hormonal status of women and in patients at four stages of autologous peripheral stem cell transplantation (APSCT)

**Serum OPN concentrations (pg/ml)**		**APSCT**			
**Controls**		**day -3/-7 (n=10)**	**day 0 (n=10)**	**day +7 (n=10)**	**day +14 (n=10)**
**Total (n=23)**	902.22 ± 395.45	3082.28 ± 2239.4	4384.92 ± 3908.47	2474.2 ± 1544.02	2844.64 ± 2346.42
**Age**					
25-34 years (n=7)	842.00 ± 299.17	1802.60 ± 1820.09	2563.40 ± 180.17	3086.40 ± 349.59	2987.80 ± 4225.39
35-59 years (n=6)	790.6 ± 319.75	2790.33 ± 2272.93	3240.90 ± 2056.10	1790.27 ± 1512.28	2985.53 ± 3374.09
60+ years (n=10)	1011.36 ± 491.36	3422.64 ± 2874.25	5228.80 ± 5619.70	2450.80 ± 1968.77	2429.68 ± 1776.75
**Sex**					
Male (n=3)	1099.87 ± 682.11	911.67 ± 632.91	1796.77 ± 763.12	1267.20 ± 1371.52	537.67 ± 583.17
Female (n=20)	872.58 ± 353.56	3764.91 ± 2331.05	5086.17 ± 4550.31	2856.7 ± 1606.59	3638.23 ± 2350.77
**Hormonal status**					
Premenopausals (n=10)	821.64 ± 332.14	3089.60	2690.80	3333.60	5975.60
Postmenopausals (n=10)	923.523 ± 84.47	3877.47 ± 2532.61	5485.40 ± 4426.02	2777.07 ± 1744.78	3248.67 ± 2314.43

APSCT (autologous peripheral stem cell transplantation); n (number of cases); osteopontin (OPN)

Table S4. Salivary osteopontin (OPN) concentration in healthy controls in relation to age, sex and hormonal status of women, and in patients at four stages of autologous peripheral stem cell transplantation (APSCT)

**Salivary OPN concentrations (pg/ml)**		**APSCT**			
**Controls**		**day -3/-7 (n=10)**	**day 0 (n=10)**	**day +7 (n=10)**	**day +14 (n=10)**
**Total (n=23)**	75.16 ± 67.79	62.64 ± 56.22	52.98 ± 86.67	204.63 ± 210.10	139.63 ± 94.63
**Age**					
25-34 years (n=7)	101.43 ± 53.9	54.40 ± 76.93	97.10 ± 137.32	333.70 ± 238.01	74.40 ± 105.22
35-59 years (n=6)	130.53 ± 41.4	94.22 ± 28.41	0.00	125.33 ± 63.98	133.93 ± 44.78
60+ years (n=10)	23.54 ± 52.93	47.00 ± 64.36	67.12 ± 94.14	200.58 ± 264.67	169.14 ± 114.63
**Sex**					
Male (n=3)	26.2 ± 45.38	78.53 ± 68.60	130.27 ± 112.82	119.37 ± 40.86	211.57 ± 96.35
Female (n=20)	82.5 ± 68.33	55.84 ± 54.70	19.86 ± 52.54	241.17 ± 245.90	108.80 ± 81.50
**Hormonal status**					
Premenopausals (n=10)	127.1 ± 39.06	0.00	0.00	502.00	0.00
Postmenopausals (n=10)	37.9 ± 62.54	65.14 ± 53.51	23.17 ± 56.75	197.70 ± 238.09	126.93 ± 65.89

APSCT (autologous peripheral stem cell transplantation); n (number of cases); OPN (osteopontin)

Table S5. Unstimulated whole saliva (UWS) flow rate in healthy controls in relation to age, sex and hormonal status of women, and in patients at four stages of autologous peripheral stem cell transplantation (APSCT)

**Unstimulated whole saliva (UWS) flow rate (ml/min)**		**APSCT**			
**Controls**		**day -3/-7 (n=10)**	**day 0 (n=10)**	**day +7 (n=8)**	**day +14 (n=9)**
**Total (n=23)**	0.64 ± 0.35	0.68 ± 0.29	0.29 ± 0.21	0.24 ± 0.19	0.23 ± 0.17
**Age**					
25-34 years (n=7)	0.83 ± 0.20	1.00 ± 0.14	0.30 ± 0.14	0.50 ± 0.00	0.38 ± 0.03
35-59 years (n=6)	0.48 ± 0.40	0.69 ± 0.37	0.37 ± 0.38	0.12 ± 0.11	0.19 ± 0.10
60+ years (n=10)	0.59 ± 0.37	0.54 ± 0.21	0.24 ± 0.13	0.20 ± 0.16	0.12 ± 0.13
**Sex**					
Male (n=3)	0.63 ± 0.36	0.77 ± 0.23	0.50 ± 0.26	0.33 ± 0.15	0.29 ± 0.12
Female (n=20)	0.70 ± 0.28	0.64 ± 0.32	0.20 ± 0.12	0.19 ± 0.19	0.15 ± 0.14
**Hormonal status**					
Premenopausals (n=10)	0.62 ± 0.33	1.10	0.20	0.50	0.36
Postmenopausals (n=10)	0.64 ± 0.40	0.56 ± 0.28	0.20 ± 0.13	0.14 ± 0.15	0.11 ± 0.11

APSCT (autologous peripheral stem cell transplantation); n (number of cases); UWS (unstimulated whole saliva)

A

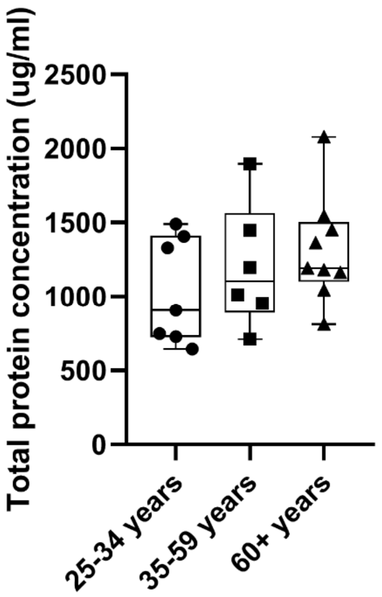

B

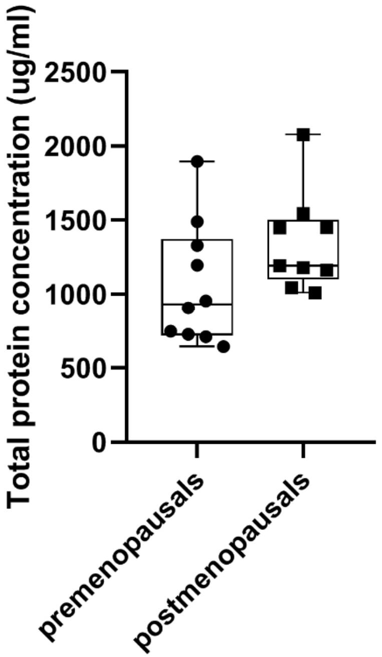

Supplementary Figure 1. Salivary total protein concentration in healthy controls in relation to age (A) and hormonal status of women (B). Values are expressed as sample means. The small black configurations (dots, triangles, squares, etc.) are individual data points, and the box plots denotes the data mean. Error bars represent the standard deviations which describe average difference between the data points and their mean.

A B

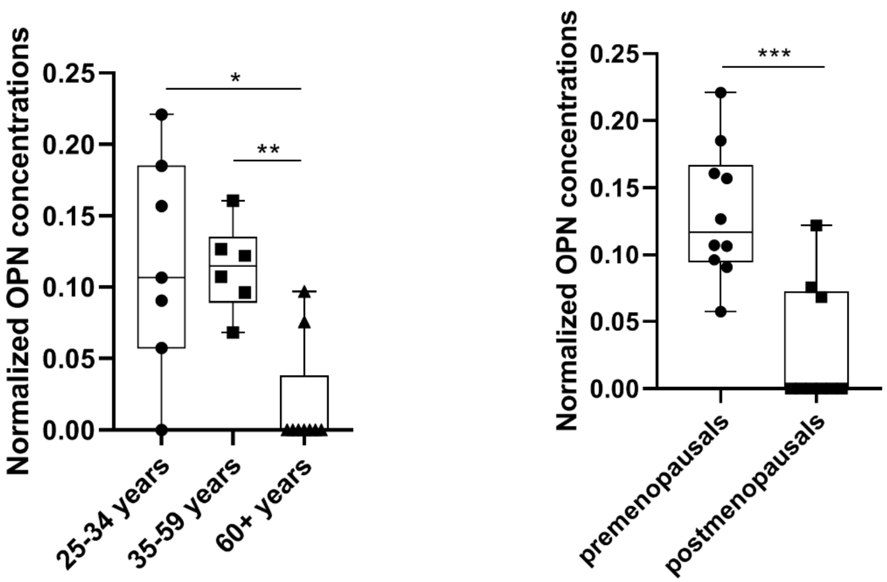

Supplementary Figure 2. Salivary normalized osteopontin (OPN) concentrations in healthy controls in relation to age (A) and hormonal status of women (B). Values are expressed as sample means. The small black configurations (dots, triangles, squares, etc.) are individual data points, and the box plots denotes the data mean. Error bars represent the standard deviations which describe average difference between the data points and their mean. (*p<0.05, **p<0.01, ***p<0.001).
